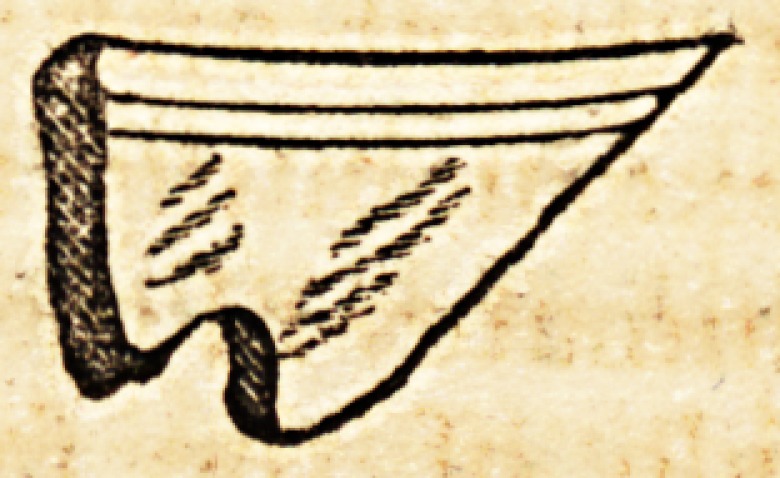# A History of a Case of Severe Neuralgic Affection of the Face, Which Was Cured by the Extirpation of a Piece of China from the Cheek, after It Had Been Imbedded in Its Substance Fourteen Years

**Published:** 1823-03

**Authors:** Henry Jeffreys

**Affiliations:** Surgeon to the St. George's and St. James's Dispensary, and Assistant Surgeon to the Lock Hospital.


**Art. III.**—*A History of a Case of severe Neuralgic Affection of the Face, which was cured by the Extirpation of a Piece of China from the Cheek, after it had been imbedded in its Substance fourteen Years.* By Henry Jeffreys, Esq. Surgeon to the St. George's and St. James's Dispensary, and Assistant Surgeon to the Lock Hospital.

The morbid affections of the nerves have, of late years, deservedly occupied a considerable share of the attention and ingenuity of medical men; nevertheless, the precise nature of these disordered actions is still involved in considerable perplexity and obscurity; nor has any mode of treating them been pointed out, which can be recommended or relied upon with confidence. The phenomena which they give rise to, present considerable variety in their character and severity, which may depend in some degree on the nature of the exciting cause on the parts affected, and on the previous state of the patient's system; but they are almost always of a very painful and distressing kind; their duration is often protracted and uncertain; and they offer a resistance to the influence of the most powerful remedies, which is not often met with in the treatment of other painful disorders.

These affections very often depend upon the application of some mechanical injury to one or more branches of a particular nerve: they are not an unfrequent consequence of the lodgment of a foreign body within the common integuments, in gun-shot and other wounds. They are also brought on by the infliction of blows on the surface of the body, by cuts, punctures, burns, &c. It is worthy of remark, that the continued application of the primary source of irritation is not necessary to the duration of the disease: once excited, it has the power of extending the disordered actions to every part of the nervous system. Thus, a puncture in the point of the finger has been succeeded, in the person of a young lady, by a train of neuralgic symptoms, of the most acute and agonizing description, which spread from limb to limb till they at length occupied the whole frame, and have now tormented her for several years.

In many cases, the invasion of this disorder cannot be traced to any evident source; a circumstance which, coupled with our imperfect knowledge of the physiology of the nervous system, will account in a certain measure for the little success which has attended almost every mode of treatment that has been proposed. In neuralgia arising from mechanical pressure on one or more nervous filaments, a better chance of rendering permanent benefit to the patient is afforded by the removal of the offending cause. In the following case, a perfect cure was effected by this means after fourteen years of acute suffering.

I do not flatter myself that it will add much to our limited stock of information on these important and interesting subjects; but it appears to be curious from the circumstances attending it, and may therefore be judged worthy of record in the pages of the London Medical and Physical Journal.

Mary Anne Asgill, twenty years of age, a country girl, with a florid complexion, and of a robust make, came under my care, on the 26th November, 1821, for a severe neuralgic affection of the left side of her face, of fourteen years' standing. She stated that, when she was six years old, she fell down while running across a room with a teacup in her hand; that the cup was broken in the fall, and the fragments made a large lacerated wound in the integuments covering the lower jaw, near to the chin; that the wound proved tedious and intractable, and was not perfectly healed in less than a year, leaving a large irregular cicatrix, which still remained; that very shortly after the occurrence of the accident she became affected with paroxysms of pain in the side of the face, of the most agonizing description, recurring at frequent and irregular intervals, and destroying her rest at night; and that she did not recollect to have enjoyed an interval of ease of twenty-four hours' duration during the whole period that she had been afflicted. She said that she had been under the care of several medical men at different times in the country, but had derived no material relief from the remedies they had recommended; that she was convinced there was a fragment of the teacup lodged in her cheek, but could never persuade any of her medical attendants to think so.

At the time of her application to me, the spasmodic paroxysms had become more frequent and severe, and the pain at night was sometimes so excruciating and intolerable, that she often disturbed the other inmates of the house with her screams. All the muscles on that side of the face were paralyzed; she could neither masticate her victuals nor laugh on that side of the mouth; the whole cheek was flattened, relaxed, and flabby; the mouth was drawn to the right side; the ala nasi of the affected side dropped more than the other, and it was not elevated during an inspiration; the opening of the nostril was smaller, and the sense of smell was less acute in that than in the other; the orbicularis palpebrarum had lost its tone, in consequence of which the under eyelid hung lower than the opposite one, exposing a larger portion of the ball of the eye than it ought to do, and preventing her from entirely closing it. The sight of that eye was considerably impaired, especially if she tried to use the organ in a strong light or by candle-light. At such times she would have severe darting pain through the bottom of the eye, accompanied with flashes of light; and a perseverance in the attempt to use it would bring on the spasms and twitchings in the rest of the face. During a paroxysm of pain, and when laughing, the cornea was spasmodically drawn under the lower lid; and she complained that the eye always felt as if it was confined and restricted in its motions.

The skin covering all the parts affected was exquisitely sensible and tender to the touch, but she referred the principal seat of her distress to a spot immediately in front of the anterior edge of the ascending ramus of the lower jaw, and nearly in the centre of the cheek. At this part a small hard point could be distinctly perceived under the integuments, which did not feel to the finger to be larger than the blunt end of a common probe. The slightest touch upon the skin of the cheek, but more especially on this particular part, brought on the spasms, and prevented an accurate examination. It was here that she thought a piece of the cup was lodged; and I strongly encouraged her in the desire to have it cut down upon, and removed. The hardness had all the feel, as well as it could be ascertained, of being occasioned by a solid body; but, whether that should prove to be the case or not, the removal of the indurated part appeared calculated to afford some mitigation of the poor girl's sufferings. In all other respects she was in perfect health, and fuller in flesh than could have been expected after so many years of pain and torture.

She took an aperient medicine on the 27th; and on the following day I cut down on the indurated part: as soon as the knife had penetrated the integuments, its edge grated against a hard substance, which, when extracted, proved to be a portion of a china teacup, of the size and shape here delineated. She suffered very acutely during the operation; but, the moment the fragment of china was removed, she expressed herself to be very much relieved from her accustomed pain.

The piece of china was unaltered in its appearance, and a gilt line upon it was quite perfect. It had lain nearly diagonally on the cheek, the broad end having been inclined forwards and inwards, and the sharp point, which was the part felt before the operation, lying backwards and outwards. The wound was dressed with adhesive plaster.

*November 30th.*—The cheek was slightly tumefied, and she complained of soreness and smarting in the wound; there was also a sense of tingling throughout the cheek. The eye, she said, felt as if it had more space and freedom to move in, and there was a smarting pain in it. There had been no return of the old spasms.

*December 5th.*—The wound was healed, with slight hardness of the cicatrix. She still complained of soreness and tenderness in the cheek; but there had been no return of the original pain, and the lower eyelid had regained its natural situation and power.

*December 8th.*—The soreness of the cheek was nearly gone. She had not ventured to eat or laugh on that side of the mouth. She said, there was a feeling about the cheek as if a heavy weight, or tight cord, had been removed from it. It was still relaxed and flabby compared with the other, but the muscles were beginning to recover their actions.

*December 18th.*—She was quite free from pain and soreness. The muscles had regained their power of action in a considerable degree, and the cheek had nearly the same plumpness and appearance as the other. The sensations were natural, and she could eat and laugh on that side without inconvenience.

*December 29th.*—There was still a sense of weakness in the eye when used upon dark-coloured needle-work by candle-light: in other respects, very little, if any, difference could be perceived between that side of her face and the other.

*January 12th,* 1822.—She had been suffering for two or three days from a return of the original pain, but in a much milder degree than formerly, and it was confined to that part of the cheek nearest the nose. It was brought on chiefly by using the eye by candle-light, and went off after keeping the lids closed for a few minutes. There was still some degree of thickening about the place of the incision. It was ordered to be kept covered with soap-cerate.

*January 20th.*—The pain had entirely left her. The cheek was as plump and as firm as the other; and the functions of eating, laughing, &c. were performed on that side without inconvenience or trouble.

I had several opportunities of seeing this patient for the two or three months that she remained in town after this, and during that period there was no appearance of her disorder returning.

In reflecting upon the circumstances of this case, I am led to believe that the piece of china, from its size, and the place it occupied in the cheek, must have pressed both upon the portio dura of the seventh, and on the facial branches of the fifth pair of nerves. It is surprising that it should have remained in such a situation for so many years, without exciting inflammation and abscess in the soft parts, or producing some more permanent disturbance in the functions or structure of the affected nerves. There had probably been no predisposition to neuralgia in the patient's system, otherwise the result of the operation might not have been so immediate and complete. In the physiological researches on the functions of the nerves which have been instituted of late years, very opposite deductions have been drawn by different philosophers from the same set of experiments; and very plausible and seducing hypotheses have only held their places till they were expelled and overthrown by the contradictory testimony of the next person who has undertaken a similar investigation to that on which their unsteady reputation was erected. Whether the case which has been related may be considered as bearing in any way upon the "symmetrical and respiratory" views of the nervous systems that have been lately promulgated, I shall leave to those more actively engaged in the inquiry to determine.

Among the modes of treatment which have been proposed and practised in these painful affections, may be reckoned the division of the disordered nerve, and the amputation or extirpation of the part in which the disease is supposed to have originated. The first of these operations has not often afforded permanent benefit; but several cases have occurred wherein amputation has been followed by a successful termination. It may appear to be a cruel alternative to propose to a patient; but experience not only justifies the practice, but it teaches us that, the earlier it is had recourse to, the more likely will it be to afford a permanent and decisive relief. The medical treatment has been, for the most part, little better than empirical, and has consisted chiefly in the almost useless exhibition of powerful and deleterious narcotics. The ferri carbonas, however, has lately been recommended on very respectable authority, and its character is supported by the result of well-attested cases, wherein it has been administered. I am now giving it to a boy of thirteen, affected with neuralgia of the branches of the median nerve, the consequence of a burn on the end of the thumb. At present the symptoms appear to be yielding to its use; and I hope, in a future Number of this Journal, to be able to record an additional testimony in favour of the new remedy.

*Clarges-street, Piccadilly; Dec. 2, 1822.*

## Figures and Tables

**Figure f1:**